# Evaluating sea cucumbers as extractive species for benthic bioremediation in mussel farms

**DOI:** 10.1038/s41598-023-28733-7

**Published:** 2023-01-26

**Authors:** Luca Grosso, Massimo Rampacci, Davide Pensa, Alessandra Fianchini, Esin Batır, İlhan Aydın, Laura Ciriminna, Pedro M. Felix, Ana Pombo, Alessandro Lovatelli, Salvatrice Vizzini, Michele Scardi, Arnold Rakaj

**Affiliations:** 1 Experimental Ecology and Aquaculture Laboratory, Department of Biology, University of Rome Tor Vergata, Via Cracovia 1, 00133 Rome, Italy; 2 PhD Program in Evolutionary Biology and Ecology, Department of Biology, University of Rome Tor Vergata, Rome, Italy; 3 A.Ge.I. Agricoltura Gestione Ittica S.C.R.L., Via Orti Della Farnesina 116, 00135 Rome, Italy; 4 Central Fisheries Research Institute, 61025 Trabzon, Turkey; 5 General Directorate of Fisheries and Aquaculture, Dumlupınar Ave., 161/1, 06800 Ankara, Turkey; 6 Department of Earth and Marine Sciences, University of Palermo, Via Archirafi 18, 90123 Palermo, Italy; 7 Faculdade de Ciências, MARE – Marine and Environmental Sciences Centre, Universidade de Lisboa, Lisbon, Portugal; 8 Departamento de Biologia Animal, Faculdade de Ciências, Universidade de Lisboa, Lisbon, Portugal; 9 MARE—Marine and Environmental Sciences Centre, Polytechnic of Leiria, 2520-630 Peniche, Portugal; 10 Fisheries and Aquaculture Policy and Resources Division Food and Agriculture Organization of the United Nations (FAO), Rome, Italy; 11 National Inter-University Consortium for Marine Sciences-CoNISMa, Piazzale Flaminio 6, 00196 Rome, Italy

**Keywords:** Ecology, Ecology

## Abstract

Filter-feeding mussels blend suspended particles into faeces and pseudo-faeces enhancing organic matter flows between the water column and the bottom, and strengthening benthic-pelagic coupling. Inside operating farms, high bivalve densities in relatively confined areas result in an elevated rate of organic sinking to the seabed, which may cause a localized impact in the immediate surrounding. Deposit-feeding sea cucumbers are potentially optimal candidates to bioremediate mussel organic waste, due to their ability to process organic-enriched sediments impacted by aquaculture waste. However, although the feasibility of this polyculture has been investigated for a few Indo-Pacific species, little is known about Atlanto-Mediterranean species. Hence, for the first time, in the present study, we conducted a comparative investigation on the suitability of different Mediterranean sea cucumber species, to be reared in Integrated Multitrophic Aquaculture (IMTA) with mussels. A pilot-scale experiment was accomplished operating within a mussel farm where two sea cucumbers species, *Holothuria tubulosa* and *Holothuria polii*, were caged beneath the long-line mussel farm of *Mytilus galloprovincialis*. After four months, *H. tubulosa* showed high survivorship (94%) and positive somatic growth (6.07%); conversely *H. polii* showed negative growth (− 25.37%), although 92% of specimens survived. Furthermore, sea cucumber growth was size-dependent. In fact, smaller individuals, independently from the species, grew significantly faster than larger ones. These results evidenced a clear difference in the suitability of the two sea cucumber species for IMTA with *M. galloprovincialis*, probably due to their different trophic ecology (feeding specialization on different microhabitats, i.e. different sediment layers). Specifically, *H. tubulosa* seems to be an optimal candidate as extractive species both for polycultures production and waste bioremediation in *M. galloprovincialis* operating farms.

## Introduction

Mussel farming provides more than 1/3 of EU aquaculture products, being one of the most spatially-extended sea farming productions in Europe^1^. It is overall considered a sustainable aquaculture industry as mussels are filter-feeders able to feed on suspended organic matter, without additional food supplies^2^. Their low-feeding requirements make the ecological footprint of mussel aquaculture much lower than that of other cultured species^3^. Moreover, mussels enhance organic matter flows between the water column and the bottom, and strengthen pelagic-benthic coupling^4^. In fact, they act as efficient collectors of particulate organic matter^2,5,6^, promoting its settling on the seabed. This organic biodeposit, although may cause a negative side effect impacting locally benthic communities^7^, may also represent an additional food source for other species tolerant to organic pollution, such as interface and deposit feeders^8^. Hence, considering the wide spatial extension of mussel farming, the integration of deposit-feeders as extractive species in these productive systems may act as an innovative and sustainable form of Integrated Multi-trophic Aquaculture (IMTA). IMTA, indeed, is based on the community-like model resulting in circular economy approach: species of different trophic levels are integrated simulating a natural community, in order to encourage nutritional recycling within aquaculture farms by the employment of extractive species able to remove and convert waste and/or uneaten feed in new biomass of commercial value^9^. In this context, sea cucumbers are among the most promising extractive species. In fact, in Asian countries, these species gain high market values being considered luxury seafood^10,11^. Moreover, deposit feeding sea cucumbers are able to ingest sediment and organic material^12–14^, playing an important role in material recycling and energy flow in benthic ecosystems^15^. Hence, they are expected to efficiently process enriched benthic sediments under farms, transforming bacterial, fungal and detrital organic matter into high-market value products^16^. On this view, the integration of sea cucumber in mussel farms may provide many advantages including increased productions through a more efficient trophic resource utilization, diversification of aquaculture products and minimization of benthic impact and deterioration.

Although sea cucumbers are promising IMTA candidates, the information about their feeding behavior and growth performance in co-culture with mussels are accessible only for a few species. While data are available for the Indo-Pacific *Australostichopus mollis* (Hutton, 1872)^17–26^, little is known about Atlanto-Mediterranean species^16,27^. *Holothuria tubulosa* (Gmelin, 1791) and *Holothuria polii* (Delle Chiaje, 1824) are sympatric sea cucumbers in Mediterranean benthic communities and important ecosystem engineers with a key role in maintaining biodiversity and health of the sediments in which they inhabit^28,29^. Furthermore, these sea cucumbers are two of the most valuable species in the Mediterranean Sea, having recently become a new fishing target^28–34^. At these latitudes, sea cucumber fishery has grown rapidly over the last decade, in the face of an increasingly interconnected and globalized market^28^. As result, their market value has increased exponentially, leading to a rapid decline or even local collapse of natural stocks. This phenomenon has stimulated the development of their aquaculture, through the recent setting up of artificial reproduction and hatchery protocols^28,29^ and feeding trials^35–37^. In addition, laboratory and pilot-scale studies have already suggested *H. tubulosa* and *H. polii* as optimal candidates for IMTA applications, both in co-culture with finfish^38–41^ and sea urchins^42^. However, to the best of our knowledge,^27^ was the sole available investigation on co-culture potential of Mediterranean sea cucumber species (*H. polii*) with mussels.

The present study aimed to expand this basis of knowledge, comparing for the first time the feasibility of Mediterranean sea cucumbers, *H. tubulosa* and *H. polii*, as extractive species in an off-shore mussel farm. More specifically, the two sea cucumber species were reared under a mussel farm for four-months to evaluate the suitability of mussel biodeposit as a food source for sea cucumbers. Survivorship and somatic growth were assessed, and size-related growth performance was evaluated by comparing two different size classes (subadults and adults) for each species. The main purpose of this study was, therefore, to evaluate the disposition of these sea cucumber species in IMTA, by understanding the difference in their feeding ecology that allow their coexistence through niche segregation in the many benthic habitats.

## Materials and methods

### Study area

**Figure 1:**
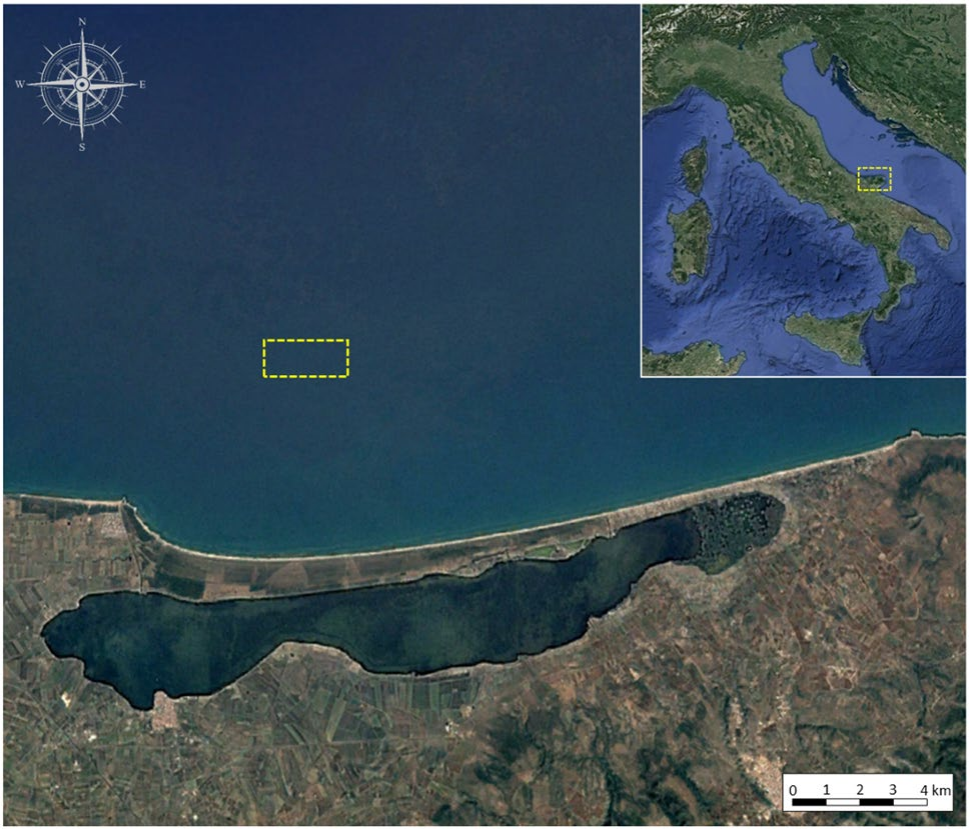
Studied area in the Adriatic Sea in Italy. The yellow dotted line signs the position of operating mussel farm under which the sea cucumber rearing cages were located. Attribution: image ©2022 Google Earth, data SIO/NOAA/U.S.Navy, NGA,GEBCO.

**Figure 2:**
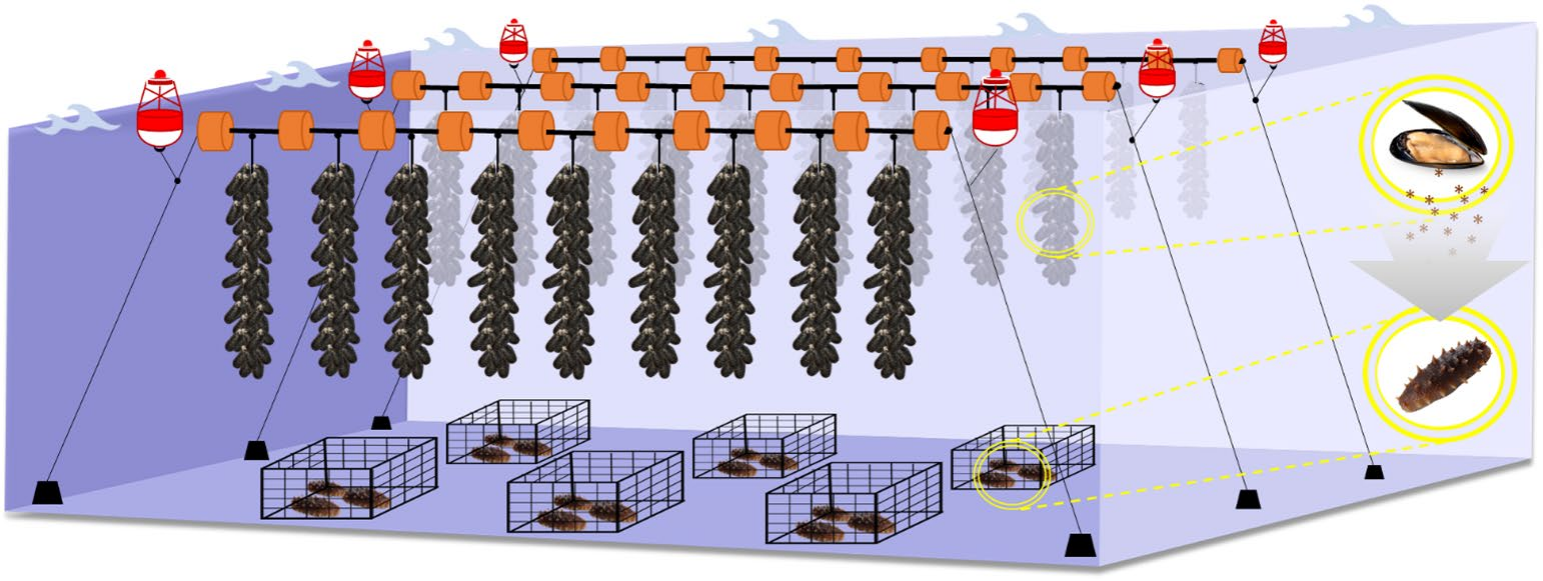
Experimental scheme of Integrated Multitrophic Aquaculture, using *Holothuria tubulosa* and *Holothuria polii* as extractive species under a mussel (*Mytilus galloprovincialis*) farm*.*

The study was carried out at Ittimar Soc. Coop. mussel farm, a *Mytilus galloprovincialis* (Lamarck, 1819) longline submerged productive system. This off-shore farm is located in the south of the Adriatic Sea, Italy (41° 58′ 55′′ N; 15° 23′ 52′′ E), 6 km from the coast (Fig. 1). The bathymetry of the area ranged from 17 to 24 m and soft sediments characterize the seafloor (Well Sorted Fine Sands). The mussel farm consists in 10 longlines spaced 70 m apart, covering a total area of 2.06 km^2^ (206 ha). Each longline is approximately 2 km long and consists of a backbone rope, supported by buoys and fixed by anchor blocks to the seabed (Fig. 2). Mussels grow up in spat-laden culture rope.

### Sea cucumbers: collection and biometric measures

Specimens of *H. tubulosa* (N = 200) and *H. polii* (N = 200) of different sizes were collected by SCUBA (Self-Contained Underwater Breathing Apparatus) divers along Apulian coast (Fasano, BR). Sea cucumbers were then transported to the mussel farm inside 500 L tanks with seawater, equipped with oxygen insuffler and dry ice (to maintain low temperature and saturated oxygen level). Once in the mussel farm facility, specimens underwent a fasting period of 48 h in lantern nets, to empty their digestive tracts. After the fasting period, sea cucumbers were sorted from the initial pool and placed for 10 min on a filtering bed, to void water from their respiratory trees^42^, before being weighed (± 0.01 g accuracy). During the measurement procedure, specimens of *H. tubulosa* and *H. polii* were sorted into two size classes (small size: 40–100 g; large size: 100–200 g).

### Experimental design and rearing systems

**Figure 3:**
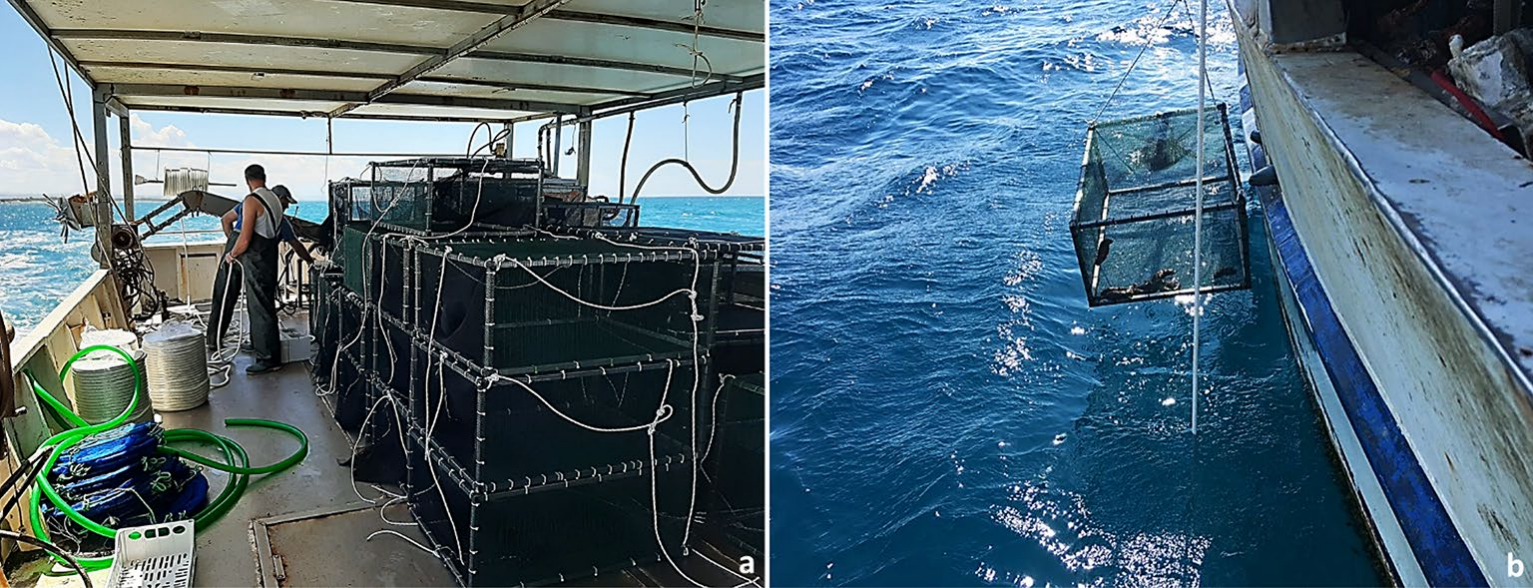
Sea cucumber experimental cages (**a**) and cage deployment (**b**).

Forty experimental cages (1 m × 0.70 m × 0.40 m, l × w × h), 20 for each sea cucumber species, were built in metal frames covered with 1 cm mesh polyethylene net (Fig. 3). For each species, 10 cages were allocated for small and 10 cages for large sea cucumbers. The initial stocking density was defined in terms of biomass (670 g m^−2^), to make growth output comparable between *H. tubulosa* and *H. polii*, since these species show interspecific differences in terms of allometry and maximal growth^43,44^.

Once at the study area, small and large *H. tubulosa* and *H. polii* were randomly assigned to two lines of 20 cages, spaced 10 m apart. These culture systems were anchored in parallel to two backbone ropes of mussel farms, following the longline orientation (Fig. 2). Moreover, to avoid the hydrodynamic drift, the cages were fixed by anchors to the seabed and partially submerged into the bottom sediment. This arrangement prevented cage displacement during severe hydrodynamic conditions and maintained sea cucumbers on the sediment below the mussel rearing nets. This pilot-scale co-culture experiment lasted four months, from 6th April to 5th August 2020, during which water temperature ranged from 18 to 26 °C.

The sea cucumber cages were regularly maintained and cleaned up to allow unrestricted biodeposit precipitation within them. Specifically, every month, SCUBA divers monitored and, when necessary, removed mussel shell fragments, fouling organisms, and sediment from the upper side of the cages.

### Biometric measurements and data analysis

At the end of the experiment, all rearing cages were recovered, and sea cucumbers biometry was performed after a fasting period of 48 h in lantern nets. For both *H. tubulosa* and *H. polii*, the survival of specimens of each size class was recorded by counting the specimens in each culture systems^17^, and their final wet weight was measured, following the standard protocol described above.

Somatic Growth Rate (SGR), Relative Weight Gain (RWG) and Growth Rate (GR) were then calculated as follows^37,41^:

- SGR (% day^−1^): ((lnW_f_ − lnW_i_)/t) × 100;
- RWG (%): (W_f_ − W_i_)/W_i_) × 100;
- GR (g day^−1^): (W_f_ − W_i_)/t;
- Survival rate (%) = 100 × (n_f_/n_i_);

where W_f_ and W_i_ are the final and initial wet weights (g) of sea cucumbers, n_f_ and n_i_ are the final and initial number of small and large sea cucumbers in all the culture systems and t represents time in days of the experiment.

### Statistical analysis

Before the analysis, raw data were diagnosed for normality of distribution and homogeneity of variance through a Levene’s test and a Kolmogorov–Smirnov test respectively^45^. Growth data were then transformed with Box-Cox transformation to improve normality and homogeneity of variances. Transformed data were close to a normal distribution (Kolmogorov–Smirnov p > 0.05) with similar variances (Levene’s p > 0.05). Chi-squared tests were performed to compare the survivorship of the different experimental groups (small and large specimens of *H. tubulosa* and *H. polii*) after four months of IMTA with *M. galloprovincialis*. Furthermore, two-way analysis of variance, ANOVA, was carried out to test the differences in terms of somatic growth rate between the sea cucumber size-classes (small and large specimens) and species (*H. tubulosa* and *H. polii*) and the interactions among these experimental groups (Table 2). When significant differences (*p* < 0.05) were generated in ANOVA, Tukey’s multiple comparison tests were used to evaluate differences among pair-wise means (*p* < 0.05). Statistical analyses were performed with PAST 3.0^46^.

## Results

At the end of the 4-month experiment, all the rearing cages were found to be well preserved. Indeed, as confirmed by SCUBA divers’ inspections during the experiment, cage setup was maintained and no damage was registered from intense hydrodynamic events. Also, the level of fouling on the upper side of the cages was successfully controlled by SCUBA divers allowing the continuous inflow of organic matter from the mussel farm.

### Survival rates

Survival rate in *H. tubulosa* and *H. polii* was overall very high, respectively 94% and 92%, with no significant differences between species (Chi-square tests: χ^2^ = 0.31;* p* > 0.05). In *H. tubulosa*, large specimens showed a higher survival rate (100%) than small specimens (90%) (Chi-square tests: χ^2^ = 4.25;* p* < 0.05), while no difference (Chi-square tests: χ^2^ = 0.02;* p* > 0.05) occurred between-size classes in *H. polii* (92% for both large and small specimens) (Table 1).

### Growth performances

**Figure 4:**
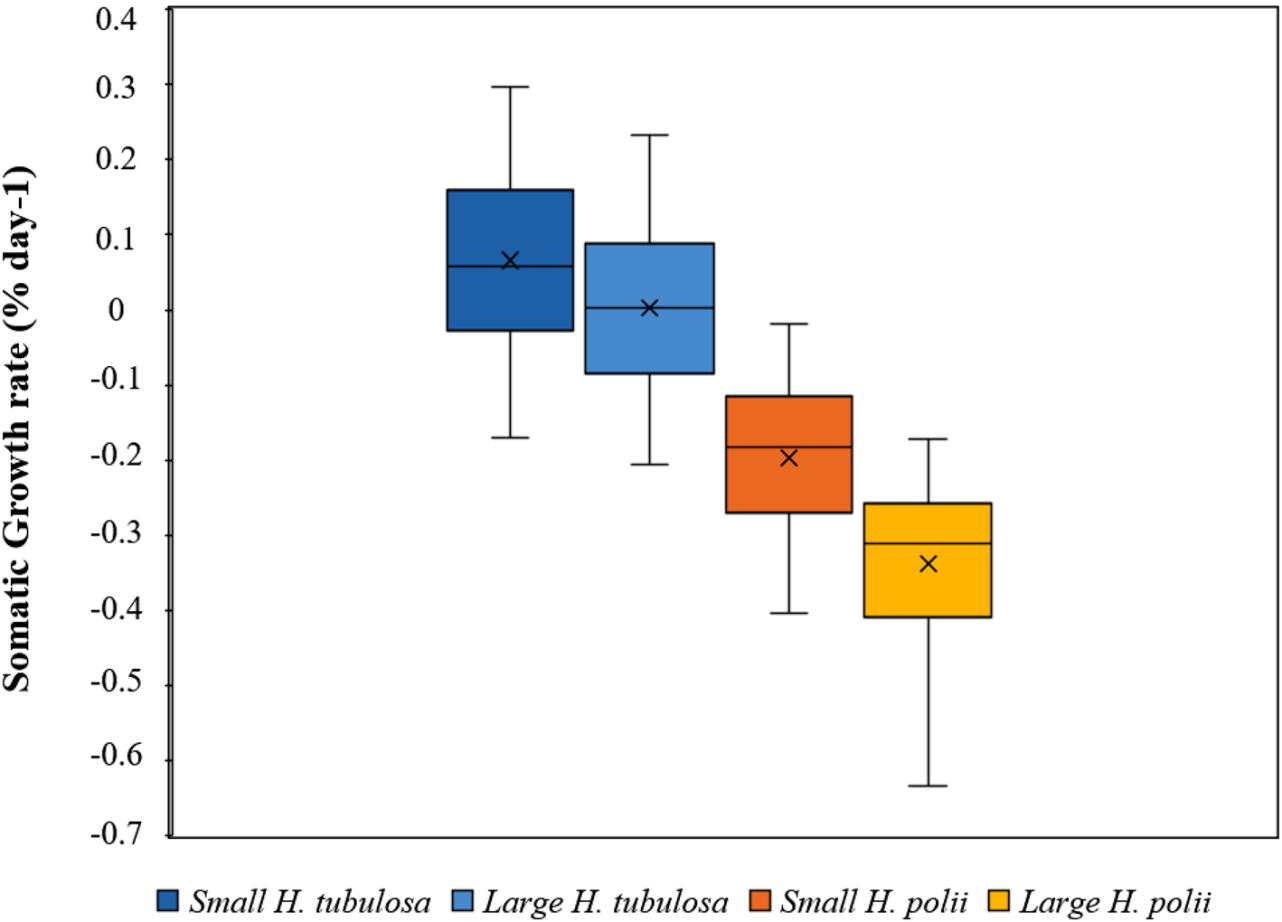
Somatic Growth Rate (SGR% day^−1^) of small and large specimens of *Holothuria tubulosa* and *Holothuria polii* reared below an off-shore mussel farm over the four-month experiment*.*

The results of ANOVA analysis showed significant differences between species and size classes, for somatic growth; also, the interaction between species and size classes were significant for growth parameters (Tables 1, 2). At the end of the four-month experiment, *H. tubulosa* showed generally a higher mean growth (6.07%) than *H. polii*, which showed a negative growth (− 25.37%). Comparing the somatic growth performances between size classes, *H. tubulosa* gained a positive value in both small and large individuals (Fig. 4; Table 1), with small specimens growing faster (9.41%, 0.07% day^−1^ and 0.06 g day^−1^ respectively for RWG, SGR and GR) than large specimens (1.05%, 0.01% day^−1^ and 0.01 g day^−1^ respectively for RWG, SGR and GR). In *H. polii* weight loss was registered in both size classes (Fig. 4, Table 1), but with large specimens decreasing more (− 32.79%, − 0.34% day^−1^ and − 0.34 g day^−1^ respectively for RWG, SGR and GR) than small specimens (− 20.42%, − 0.20% day^−1^ and − 14 g day^−1^ respectively for RWG, SGR and GR) (Fig. 4, Table 1).

**Table 1:** Biometric measurements, growth performances and survival rates of small and large specimens of *Holothuria tubulosa* and *Holothuria polii* reared below an off-shore mussel farm over the four-month experiment.

	*H. tubulosa*		*H. polii*	
	Small	Large	Small	Large
Wi (g)	77.71 ± 2.06^a^	120.32 ± 2.86^b^	77.75 ± 2.04	122.64 ± 3.05^b^
Wf (g)	84.23 ± 2.65^a^	120.69 ± 2.76^b^	61.17 ± 1.55^c^	81.65 ± 2.36^a^
SGR (% day^−1^)	0.07 ± 0.02^a^	0.01 ± 0.02^b^	− 0.20 ± 0.01^c^	− 0.34 ± 0.02^d^
GR (g day^−1^)	0.06 ± 0.01^a^	0.01 ± 0.02^b^	− 0.14 ± 0.01^c^	− 0.34 ± 0.02^d^
RWG (%)	9.41 ± 2.18^a^	1.05 ± 1.97^b^	− 20.42 ± 1.26^c^	− 32.79 ± 1.37^d^
Survivorship (%)	90.00^a^	100.00^b^	91.67^ab^	92.50^ab^

*Wi* initial wet weight (g), *Wf* final wet weight (g), *SGR (% day*^*−1*^*)* Somatic Growth Rate; *GR (g day*^*−1*^*)* growth rate, *RWG (%)* relative weight gain (Means ± SE).

The different letters indicate significant differences (*p* < 0.05) among the experimental diets (two-way ANOVA followed by Tukey’s test for comparisons of Wi, Wf, SGR and RWG; Chi-squared test for comparisons of survivorship).

**Table 2:** Two-way ANOVAs, Levene’s statistic and Kolmogorov–Smirnov (K-S) statistic on somatic growth rate (SGR) of sea cucumbers, reared under a mussel farm, between species (*Holothuria tubulosa* and *Holothuria polii*), size (small and large) and their interaction [n.s: p > 0.05; *p < 0.05; **p < 0.01; ***p < 0.001].

Two-way ANOVA	SS	MS	F	p	df	Levene's statistic	p (Levene’s)	K-S statistic	p (K-S)
Species	4.88	4.88	330.50	8.98E^−43^ (***)	1	0.68	n.s	0.98	n.s
Size	0.49	0.49	32.94	3.14E^−08^ (***)	1	0.07	n.s	0.98	n.s
Interaction	0.14	0.14	9.82	0.002 (**)	1				
Within	2.69	0.01			182				

*SS* sum of squares, *MS* mean squares, *F* F values, *p* p-values, *df* degrees of freedom.

## Discussion

This study evaluated the feasibility of the co-culture between the filter-feeder *Mytilus galloprovincialis* and two species of deposit-feeder sea cucumbers (*Holothuria tubulosa* and *Holothuria polii*). As far as we know, this is the first investigation on co-culture of sympatric European sea cucumbers in association with bivalve productive farms. Bivalves possess a highly efficient filtering apparatus, which enable them to concentrate a large amount of phytoplankton and other suspended particulate matter, rejecting the undigested materials in the form of mucus-bound aggregates^47,48^. The high nutritional value of the ingested food is reflected in their metabolic wastes, and the mussel predigestion activity has been suggested to improve the nutrient bioavailability for deposit-feeder sea cucumbers^18^. Moreover, these bio-deposits, covered by a polysaccharide matrix, become largely colonized by bacteria which in turn may represent a high nutritional food source for sea cucumbers.

Survivorship is one of the main indicators of co-culture viability, evidencing potential side effects of co-existence in the same rearing environment and food source suitability in an IMTA system^42^. The present study evidenced a high survival rate of both sea cucumber species (*H. tubulosa* and *H. polii*), reflecting their compatibility with the rearing conditions experienced below an off-shore mussel farm. However, although there were no significant differences among experimental groups except for *H. tubulosa*, different survivorship trends emerged between species and size classes. Indeed, survival rate of large specimens was higher in *H. tubulosa* than in *H. polii* suggesting a different ecological compatibility of the two species with productive conditions experienced. On the other hand, considering the small size class, *H. polii* achieved a higher survival rate than *H. tubulosa*. These results may rely on a different predation susceptibly of small specimens of the two sea cucumber species. In fact, *H. polii* presents a thick body wall that may work as an efficient defense to predators^49,50^. Conversely, small specimens of *H. tubulosa* are characterized by a thinner body wall that probably makes them more vulnerable to predation by benthic organisms, such as crabs and sea stars, which were found in sea cucumber rearing cages (authors’ personal observation).

From this four-month experiment did not emerge a significant relationship between survivorship and somatic growth yield. Although survival rates were high regardless of species and size classes, growth performances ended up being significantly different between the size classes and species and from interactions between experimental groups (Table 2). These results could be related to the duration of the experiment; indeed, the sea cucumber are able to thrive without feed for several months. However, when these organisms are exposed to a suboptimal food supply for a long time, the net health outcome is negative, with a clear decrease in body size over time suggesting tissue self-digestion^51^. Therefore, the final somatic growth results confirmed a different compatibility between sea cucumber species in co-culture with mussels, as already suggested by the survivorship trend. *H. tubulosa* and *H. polii* showed opposite values of somatic growth yields. Small and large specimens of *H. tubulosa*, indeed, increased in weight over the experimental time. In contrast, *H. polii* specimens of both size classes showed a general weight loss, although reared under the same experimental conditions as *H. tubulosa*. These findings evidenced that *H. polii* presents compatibility constraints in co-culture with mussels, while *H. tubulosa* seems to be a promising extractive species. The difference in growth yields could be related to the different trophic ecology of sea cucumbers. In fact, Ref.^52^, through a stable isotope investigation, suggested a species-specific feeding traits allowing co-existence through trophic niche segregation. The niche segregation among these sea cucumbers can be explained by the differences in digestive physiology (*e.g.*, in terms of intestinal microbiota) or feeding microhabitat (*e.g.*, different sediment layers). Moreover, Ref.^53^ evidenced that *H. tubulosa* and *H. polii* adopt different feeding strategies to compensate the poor-quality feeding conditions often encountered in the wild. *H. tubulosa* actively selects organic matter from the surface sediment layer, capturing and retaining with the oral tentacles organic-rich particles^54–56^. This specific feeding strategy may explain the *H. tubulosa* capacity to efficiently exploit organic-rich mussel biodeposit as food sources. Conversely, *H. polii* is able to dig into the sediment in order to feed on deeper sediment layers. The burrowing behavior of *H. polii* may not be compatible with the physico-chemical factors below the mussel farms (e.g., reduced oxygen availability, fine sediment granulometry, excessive organic sedimentation, excessive shell drop, nitrate release^17^), that potentially affected the growth and survival of this sea cucumber species.

Significant growth performance differences also emerged between size classes of both sea cucumber species. The somatic growth values obtained for small specimens were higher than that for large ones, for both *H. tubulosa* and *H. polii*. This matches with the life history traits of echinoderms for which a progressive increase in size after the metamorphosis leads to a consequent variation in food energy allocation. In fact, the allocation is primarily somatic in small specimens and becomes gonadal in large ones, showing a strong relation with animal size and age^57^. Sea cucumber growth rates should be compared with caution among different studies due to the different species employed, size variations, rearing densities and conditions^17^. In wild, sea cucumbers are considered slow-growing organisms, since they are ectotherms that show an active regulation of energy allocation in response to the poor food quality and quantity in benthic communities^58,59^. In fact, in these habitats, sea cucumbers employ more energy for maintaining metabolic activities than for growth^60^. Conversely, when the food sources do not represent a constraint, such as in aquaculture conditions, the growth performances result significantly higher^61^. In the present study, the highest growth rates detected in small *H. tubulosa* resulted comparable with those of other IMTA investigations with bivalves in the Indo-Pacific area. During our four-month experiment, small *H. tubulosa* showed average SGR and GR respectively of 0.07% day^−1^ and 0.06 g day^−1^, with high intra‐specific variations (SGR up to 0.30% day^−1^ and GR up to 0.14 g day^−1^) that could be explained by genetic variability^62,63^. Similar values of GR were obtained in *Apostichopus japonicus* (Selenka, 1867) co-cultured with scallops (0.09–0.31 g day^−1^,^47^) Northern China; for *Australostichopus mollis* (Hutton, 1872) cultured beneath an operating green-lipped mussel farm (0.06 g day^−1^^17^) in New Zealand; for *Apostichopus californicus* (Stimpson, 1857) in polyculture with the Pacific oyster *Magallana gigas* (0.06–0.16 g day^−1^, Ref.^64^ in Canada. In the Mediterranean Sea, the growth rate in IMTA with bivalves were investigated only for *H. polii*^27^. This study, which used similar size class sea cucumbers (59 ± 10.7 g) with ours, found a negative growth in this species after a four-month co-culture with mussels and fin-fishes. Conversely, Ref.^41^, which employed juvenile *H. polii* (24.6 ± 2.1 g), evidenced positive SGR (0.18–0.2% day^−1^) during co-culture experiment under fish cages, although high mortality rates were detected in all experimental conditions. For *H. tubulosa* growth performances were only evaluated in co-culture with fin-fishes^38,40^ and sea urchins^42^. Reference^38^ recorded an SGR of 0.32% day^−1^ for *H. tubulosa,* belonging to similar size-class (92.81 ± 2.29 g) than that employed in small size class, after 90 days of rearing below commercial fish cages*.* In these investigations, the growth rate was higher than our values. However, we obtained higher growth performances than^40^, who detected a weight decrease in *H. tubulosa* (171.4 ± 64.3 g) reared in IMTA near marine fish cages. This could be related to the organic matter enrichment which is expected to highly depend on specific IMTA conditions and to the stocking densities. In the present study, the rearing density (670 g m^−2^) was lower than that in the study by^40^ (1700 g m^−2^), but higher than that in Refs.^38,41^ (respectively of 313 g m^−2^ and 253 g m^−2^). Exceeding optimal stocking densities, although it might improve organic matter bioremediation, may compromise sea cucumber somatic growth yield or time to reach the commercial size^17^. Several studies highlighted that high density could affect growth in aquatic organisms in several aspects, crowding stress (reducing water quality, competition for a limited food supply, social interactions/physical contact, chemical mediation and genetic factors)^65–68^. These factors, individually or together, could have hindered the growth rates of sea cucumbers in our study. Therefore, future investigations on adequate stocking densities for IMTA systems are essential to ensure sea cucumber survival, growth and welfare.

To summarize, the present study highlighted the potential of integrated aquaculture between mussels and sea cucumbers in the Mediterranean Sea. This polyculture is potentially able to increase energy-use efficiency, promoting productivity increase, rearing diversification and adding value to the aquaculture sector, with new commercially important species, and sediment bioremediation. Moreover, this is the first study that compared simultaneously two co-existing sea cucumbers as extractive species in a mussel farm. At the end of the four-month experiment, *H. tubulosa* and *H. polii* showed different growth performances when cultured below an offshore mussel farm. This result suggests that these species, although belonging to the same functional group, rely on species-specific feeding behavior which probably allows them to co-exist by segregating their trophic niches.

In conclusion, species that share similar life-history traits and habitat type association do not necessarily converge on feeding and rearing requirements, indeed, this is often the reason for their niche segregation^69^. Therefore, targeted studies on species ecology are necessary to develop an effective community-like aquaculture and to evaluate the compatibility of species with physico-chemical conditions (hydrodynamics, temperature, oxygen, nitrogen concentration), biochemical composition of potential food sources, aiming at the development of a fully functional bioceonosis within a IMTA farm. Further studies are needed to deeply investigate the ability of sea cucumbers to assimilate mussel waste by means of biochemical tracers such as stable isotopes and fatty acids. Specifically designed experiments must be carried out to assess the potential of Mediterranean sea cucumber species for sediment bioremediation and to define the critical biomass and carrying capacity in mussel farms.

## Data Availability

The original contributions presented in the study are included in the article. Further inquiries can be directed to the corresponding author.
